# The feasibility of implementing and evaluating individualised digital prehabilitation prior to major elective surgery in adults aged ≥ 50 years: the PreActiv intervention

**DOI:** 10.1186/s13741-026-00663-8

**Published:** 2026-03-03

**Authors:** Annabelle Emery, Rebecca Allam, David Quinn, Helen Sims, Ian-Ju Liang, Bruno Spellanzon, Julia Groot, Adam Aspbury, Harriet Bullough, Leigh Ingham, Justine Archman, Frankie Brown, Oliver J. Perkin, Alec Snow, Max J. Western

**Affiliations:** 1 Snow Squared Ltd, Brunel House 11 The Promenade, Bristol, BS8 3NG UK; 2 Royal United Hospitals Bath NHS Foundation Trust, Combe Park, Bath, BA1 3NG UK; 3 School of Medicine, University of Dundee, Dundee, DD2 9SY UK; 4 Centre for Motivation and Behaviour Change, University of Bath, Bath, BA2 7AY UK; 5 Sports Exercise and Health Science Research Group, Edinburgh Napier University, Edinburgh, EH11 1PA UK; 6 Centre for Nutrition and Exercise Metabolism, Department for Health, University of Bath, Bath, BA2 7AY UK; 7 North Bristol NHS Trust, Bristol, BS10 5NB UK

**Keywords:** Prehabilitation, Preoperative, Exercise, Health optimisation, Digital, Telehealth, Teleprehabilitation, Major surgery, Elective surgery

## Abstract

**Background:**

Prehabilitation interventions prepare patients for surgery to improve postoperative outcomes such as speed of discharge and recovery time. Although more efficient and desired than centre-based, home-based prehabilitation is often ineffective due to poor adherence and its non-tailored nature. Digital technology may improve adherence to and effectiveness of home-based prehabilitation. The present study investigated the feasibility of digital, individualised, dynamic prehabilitation (‘PreActiv’) to determine whether a definitive study is viable.

**Methods:**

In this single-arm study, *N* = 35 patients aged ≥ 50 years awaiting major elective surgery in ≥ 10 weeks were allocated to receive six weeks of digital exercise prehabilitation. Thrice-weekly exercise sessions included aerobic and resistance interval training, tailored to mobility and adapted according to repeat assessments of fitness within the digital platform. Primary outcome measures were retention (completing follow-up measurements) and adherence (completing all exercise sessions), assessed against Stop criteria ≤ 50%. Secondary outcome measures were compliance (to exercise intensity, type, duration), and acceptability, plus signal of effect, evaluated through pre- to post-intervention changes in cardiorespiratory and functional fitness, quality of life, mood, and typical physical activity.

**Results:**

Retention rate was 69%. Exercise adherence was 88% in study completers. Exercise was performed with high compliance, as the correct intensity, type, and duration was completed in 79% of sessions. All participants reported that the exercise videos were easy to follow and they would recommend the programme. Participants expressed concern when completion of prehabilitation did not coincide with surgery date. In-person measurement visits posed a barrier to recruitment and retention. Functional fitness, quality of life, and mood improved from pre- to post-intervention. At the group level, cardiorespiratory fitness was unchanged, but there were individual clinically-meaningful improvements. There were no related serious adverse events.

**Conclusions:**

A definitive study investigating the effects of this novel digital prehabilitation programme on postoperative outcomes is feasible based on achieving predefined progression criteria, and warranted based on high compliance and acceptability. Modifications to the study design such as tailoring programme length to surgery date and removing in-person measurement visits should be implemented to optimise recruitment, retention, and participant satisfaction.

**Trial registration:**

The study was prospectively registered (NCT06137781).

**Main text**.

## Background

In the National Health Service (NHS) of the United Kingdom, 1.5 million major surgeries are performed per year, costing £5.5 billion (Abbott et al. 2017). Characterised by long operative duration, haemodynamic instability, significant blood loss, and organ ischaemia (Martin et al. 2020), major surgeries are commonly accompanied by postoperative complications, with an average rate of ~ 30%, ranging from < 10% (breast surgery) to > 50% (cardiac surgery) (International Surgical Outcomes Study group 2016). The most prevalent complications are postoperative bleed, arrhythmia, and infection of the surgical site, and the most serious complications are cardiac arrest, acute respiratory distress syndrome, and myocardial infarction (International Surgical Outcomes Study group 2016). Complications can negatively impact the patient’s quality of life for at least 12 months postoperatively (Downey et al. 2023). Furthermore, postoperative complications double the cost of surgery (Zogg et al. 2018, Haidar et al. 2023) and length of stay in hospital (International Surgical Outcomes Study group 2016) compared to surgeries without postoperative complications. The risk for hospital-acquired infection, poor mental health, and reduction in mobility and activities of daily living increases with length of hospital stay (Rojas-García et al. 2018). Furthermore, extended hospital stays reduce hospital bed availability, which is a prominent cause of elective surgery cancellation (Koushan et al. 2021) and prolonged waiting time for surgery, which itself is associated with anxiety, poorer perceptions of health (Oudhoff et al. 2007), morbidity, and mortality (Gibbs et al. 2024).

Prehabilitation describes interventions in the preoperative period to enhance physiological reserve for withstanding surgical stressors, improving postoperative outcomes and recovery (Fleurent-Grégoire et al. 2024). Exercise training is a cornerstone of prehabilitation due to the increased risk of postoperative complications for patients with lower cardiorespiratory fitness (Moran et al. 2016) and physical function (Moran et al. 2016). It has been shown that performing high-intensity interval training prior to major surgery improves cardiorespiratory fitness, with the potential to reduce the risk of postoperative complications by more than 50% (Clifford et al. 2023). Furthermore, multimodal prehabilitation – incorporating exercise, nutritional guidance, psychological optimisation, and breathing training – prior to major surgery can reduce the risk of postoperative complications by up to 67% and length of hospital stay by up to four days, compared to usual care (McIsaac et al. 2022).

Two-thirds of patients indicate a preference to complete prehabilitation programmes at home (Gurunathan et al. 2023, Waterland et al. 2021) due to lack of time and difficulty travelling to facilities on a regular basis posing barriers to engagement with in-person prehabilitation programmes (Gurunathan et al. 2023, Waterland et al. 2021, van der Velde et al. 2023). Conversely, opportunities for social interaction and support from healthcare professionals within supervised programmes are considered as important facilitators to engage with prehabilitation (Velde et al. 2023). Despite a majority preference for home-based prehabilitation, it has been shown that adherence and efficacy is lower for home-based compared to supervised prehabilitation programmes (Thomas et al. 2019). Together, these findings indicate that current provisions for prehabilitation do not meet the needs of patients.

There is growing interest in the use of digital technology – including video calls, apps, and wearable devices – to support the delivery of prehabilitation (Pedersen et al. 2023). In the setting of musculoskeletal physiotherapy, it has been shown that adherence to home-based exercise is superior when delivered via a digital app compared to paper handouts (Lambert et al. 2017). Furthermore, digital solutions provide opportunities for individual tailoring, objective progression, and ongoing monitoring of exercise programmes, which are identified as facilitators to engaging with prehabilitation by patients and their carers (van der Velde et al. 2023, Agasi-Idenburg et al. 2020). Digital delivery has the potential to improve accessibility, scalability, and cost-effectiveness of prehabilitation programmes, which is relevant in light of the NHS England (2023) perioperative mandate for widespread implementation of prehabilitation, and estimates that 60% of people living in England will require surgery during their lifetime (Watson et al. 2024).

In response to this unmet need, a novel digital prehabilitation programme (‘PreActiv’ (Snow Squared Ltd 2024)) which uses logic to create individualised and dynamic prehabilitation programmes based on health data, fitness test results, and patient feedback has been developed. Rigorous evaluation of ‘PreActiv’ is required prior to consideration of clinical implementation within the UK NHS. As such, a preliminary study was implemented to assess the feasibility and acceptability of ‘PreActiv’, using predefined progression criteria to evaluate whether a definitive study assessing the effects on postoperative outcomes is indicated.

## Methods

### Study design

This single-arm study included measurements before and after a digital exercise prehabilitation intervention. Measurement visits took place at the University of Bath, with participants recruited at the Royal United Hospital Bath NHS Foundation Trust. Participants received usual care throughout the study. The protocol was approved by the NHS Research Ethics Committee and Health Research Authority (reference 24/WA/0007, 25th January 2024) and the study was prospectively registered (NCT06137781).

### Recruitment

Participants were recruited via the preoperative assessment clinic at the Royal United Hospital Bath NHS Foundation Trust. Potentially eligible patients were identified from the preoperative assessment clinic booking list, and recruitment materials were sent via email to those who had ≥ 48 h prior to their appointment to consider the information. When patients attended their routine preoperative assessment appointment, they were asked if they would like to participate by a member of the preoperative team, and only those willing to participate were introduced to a member of the research team.

### Participants

Thirty-five participants aged ≥ 50 years and scheduled for major elective surgery (NICE 2020) in ≥ 10 weeks were recruited. Participants were screened by a member of the research team who was an NHS physician according to exclusion criteria: (i) presence of contraindications to maximal intensity exercise (Fletcher et al. 2013), (ii) unsuitable to increase physical activity level identified by Physical Activity Readiness Questionnaire, (iii) poorly-controlled lung conditions, diabetes, or seizures, (iv) hospital admission for cardiac issue within prior 12 months, (v) ongoing wounds or infections, (vi) unable to access technology required to engage with the intervention, (vii) currently exceeding World Health Organisation (WHO) physical activity guidelines of at least 150 min/week moderate intensity, or 75 min/week vigorous intensity, or an equivalent combination of moderate-vigorous intensity aerobic activity plus twice-weekly muscle strengthening exercise, (viii) unable to understand explanations and/or provide informed consent, (ix) unable to understand written or spoken English, and without ongoing access to an interpreter, (x) any condition and/or behaviour that would pose undue personal risk or introduce bias into the study, and (xi) currently enrolled in another research study. Participants provided written informed consent in the presence of an NHS physician.

### Intervention

**Fig. 1:**
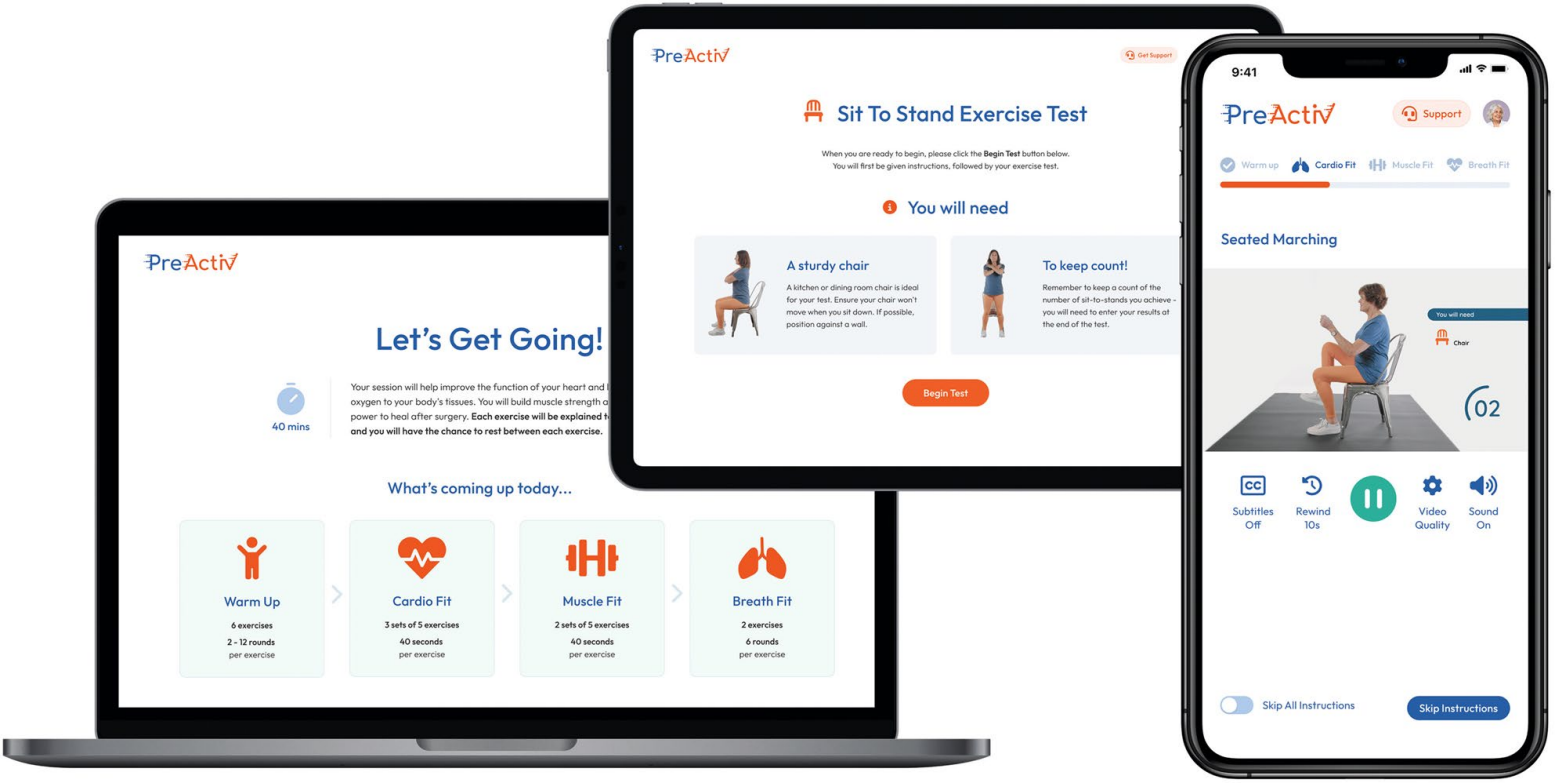
Sample screenshots of the PreActiv digital prehabilitation platform

The exercise prehabilitation intervention was delivered via a digital platform (PreActiv v1.0, Snow Squared Ltd., London, UK) which is accessible via web-browser on computers, laptops, tablet devices, and smartphones (Fig. 1). The platform hosted 18 preoperative exercise sessions, with a recommended frequency of three sessions per week for six weeks. Each exercise session was ‘unlocked’ once the previous session had been completed, and remained available until completion. A prehabilitation eBook was available within the platform which contained advice on optimising nutrition, mental health, sleep, and oral hygiene, reducing smoking and alcohol intake, and managing pain. The platform also included a community forum to allow participants to connect with each other for social support. Technical support was available to participants via an online ticketing system, and a telephone helpline was available if participants required support with their programme from a prehabilitation professional.

Participants enrolled onto the digital exercise prehabilitation platform using their personal email address during the baseline measurement visit. A researcher checked that the participant was able to log-in and hereafter all platform use was unsupervised. When participants first accessed the digital prehabilitation platform, they were presented with a welcome video explaining the structure of the prehabilitation programme and safety precautions, and participants could not proceed without viewing the video. Next, participants answered a series of health questionnaires including questions to ascertain their mobility level and allocate them suitable exercises ranging from seated to floor-based. Next, a baseline fitness assessment was completed unsupervised within the online platform, and interpreted in relation to previously published data for 1-minute sit-to-stand test (Strassmann et al. 2013) or 1-minute seated push-up test (Poncumhak et al. 2022) to inform the level of the programme. Fitness assessments were repeated after completing six, 12, and 18 exercise sessions (approximately every two weeks) to generate dynamic programmes that progressed or regressed according to objectively measured fitness level.

**Fig. 2:**
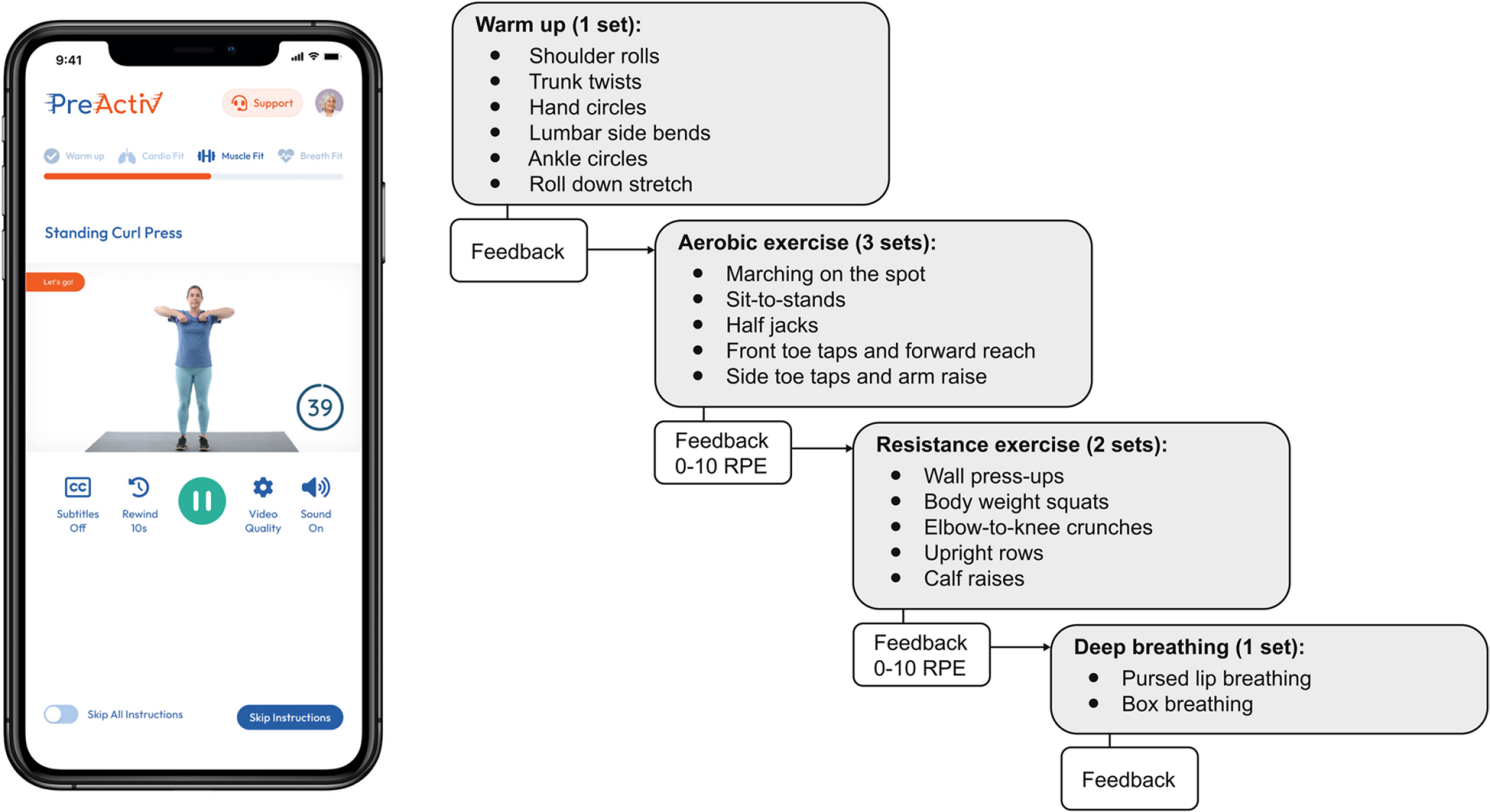
Schematic showing an example exercise session in PreActiv RPE = Rating of perceived exertion

Each exercise session was 35 min long and comprised a warm-up, aerobic exercise, resistance exercise, and deep breathing exercises. Prior to initiating this study, patient and public involvement and engagement (PPIE) informed shortening exercise sessions from 60 min to 35 min, to improve accessibility and capacity to complete three sessions per week around other commitments including medical appointments. Best practice guidelines for prehabilitation recommend including aerobic, resistance, and inspiratory exercises that are tailored to the individual in each session (Tew et al. 2018). In particular, exercise performed at higher intensities is recommended to maximise fitness gains over a short period of time before surgery (Franssen et al. 2022). All exercises were delivered via video footage of an age-appropriate model completing the exercise, and contained verbal prompts and encouragement. Detailed instructions on how to perform each exercise were available to view before the first set of each exercise, if required. Exercises were performed using the participant’s body weight or household items (chair, wall, countertop surface, and homemade weights e.g., water bottles). The warm-up consisted of gentle, whole-body dynamic mobility exercises. Aerobic exercise was three sets of five 40-second vigorous intensity intervals performed at rating of perceived exertion (RPE) ≥ 5 on a 0–10 scale (Arney et al. 2019, Garber et al. 2011), interspersed with passive rest (20 s between exercises, 50 s between sets). Resistance exercises followed aerobic exercise, and were prescribed as two sets of five 40-second exercises at RPE ≥ 5/10, each separated by 20 s passive rest, and with each set separated by 50 s rest. Resistance exercises targeted all major muscle groups and varied every six sessions to provide novel stimuli. Each exercise session concluded with one set of two deep breathing exercises, each performed for three 30-second repetitions separated by a five-second rest. An example exercise session is shown in Fig. 2.

Each completed exercise session was recorded in the participant’s profile. Participants were asked to provide feedback on their exercise session (described in detail in ‘Feasibility measurements - compliance’). All data entered into the platform were securely stored in a relational database management system and were transferred to the digital prehabilitation platform to be viewed via secure sockets layer and transport layer security encryption. Participants were only able to view their own data, and the technical and prehabilitation teams were able to view all participant data as required to provide support. In addition to providing support as requested by participants, proactive support was provided to participants if they had not engaged with the platform for four days to facilitate adherence, or if they reported an issue with specific exercises or exercise intensity to promote compliance. Wearable devices were not used as an intervention support or measurement tool.

### Feasibility measurements

#### Recruitment

Uptake was defined as the proportion of invited patients who were willing to be screened. Screen-pass rate was defined as the proportion of patients willing to be screened who passed screening. Recruitment rate was defined as the proportion of patients invited to participate who provided informed consent.

#### Retention

Retention was calculated as the proportion of participants who provided written informed consent that attended the final measurement visit for the study. In the context of prehabilitation research, follow-up measurements may not be completed due to the unexpected expediting of surgery, so retention rate was also reported considering those who withdrew for this reason to have completed the study, i.e., they have completed the clinical phase during which this research is relevant.

#### Adherence

Each time a participant completed an exercise session – by watching all exercise videos to completion (where it was not possible to skip or fast-forward) and submitting feedback question responses – an electronic record was saved to their profile, which was used to assess adherence. Adherence was defined as the proportion of sessions offered that were completed. Participants were considered ‘adherent’ if they completed 100% of available exercise sessions. As exercise sessions were independently scheduled by participants and had no expiry date, adherence to the recommended frequency of three sessions per week was evaluated by calculating the number of days to complete three consecutive sessions from a rolling average.

#### Compliance

Compliance was assessed hierarchically, with sessions first checked for intensity compliance, then exercise type, and finally duration, with non-compliance recorded at the first unmet criterion. Firstly, compliance to vigorous intensity exercise was assessed by participants self-reporting RPE immediately after aerobic training and again immediately after resistance training using a digital 0–10 RPE scale which saved their response to the online platform. A session was deemed non-compliant if aerobic and/or resistance sessions were conducted at RPE < 5/10. Secondly, compliance to the type of exercise movements prescribed was assessed by participants self-reporting any exercises they were unable to complete in a post-session feedback form. A session was deemed non-compliant if participants flagged problematic exercise(s). Thirdly, compliance to the duration of exercise was assessed by participants self-reporting how much of the session they were able to complete: All / Most / Some / None. A session was deemed non-compliant for duration if a participant reported Most / Some / None, without highlighting problematic exercise(s).

#### Acceptability

Participants completed an anonymised digital feedback form at the end of the intervention which was scored using a Likert scale: 1 - Strongly disagree / 2 - Disagree / 3 - Unsure / 4 - Agree / 5 - Strongly agree / 0 - Not applicable. Participants were also given the opportunity to provide open-ended written feedback on their overall experience. Quotes from participant feedback were thematically analysed using an inductive approach.

#### Safety

The incidence, severity, and relatedness of adverse events was categorised by NHS physicians according to Common Terminology Criteria for Adverse Events guidelines (v5.0). Adverse events were detected via patient-report in a feedback form completed after each exercise session, followed-up with a symptom review via telephone. No examinations or investigations were conducted for the purpose of assessing adverse events, but clinically recorded results were used to categorise serious adverse events.

### Pre- and post-intervention measurements

Measurements were conducted at the University of Bath by an exercise physiology researcher pre-intervention in week 0 and post-intervention in week 7. All measurements were completed before surgery.

#### Patient-reported outcomes

Participants self-reported their demographics (age, sex, ethnicity), comorbidities, medications, and surgery type. Typical physical activity volume was estimated using the International Physical Activity Questionnaire (IPAQ) short-form (Craig et al. 2003). Data were truncated and metabolic equivalent of task (MET)-hours/week calculated, as recommended (Sjostrom et al. 2005). When participants responded ‘Not sure’ instead of reporting a numerical value of days and minutes/day of physical activity, MET-hour/week calculations were not possible, resulting in missing data for *n* = 1–4 participants. Health-related quality of life was measured using the EuroQoL EQ-5D-5L questionnaire and visual analogue scale (Herdman et al. 2011). Mental health was measured using the Hospital Anxiety and Depression Scale (HADS) (Zigmond and Snaith 1983).

Participants were asked to supply information about their usual transportation to the hospital, to allow estimation of carbon emissions avoided by partaking in a digital vs. hypothetical hospital-based prehabilitation programme. For participants who usually travelled by bus, walking, or cycling, carbon emissions were considered to be zero. Participants who travelled by car were asked to report the total return journey distance from their home to the Royal United Hospital Bath NHS Foundation Trust, plus details about their vehicle to estimate carbon emissions (UK Government n.d.).

#### Resting observations

Body mass was measured using electronic scales and standing height was measured using a stadiometer. Resting heart rate, and systolic and diastolic blood pressure were measured in triplicate after 15 min of seated rest using an automated sphygmomanometer, with the average reported.

#### Cardiorespiratory fitness

Cardiorespiratory fitness was measured using a ramp-incremental cardiopulmonary exercise test (CPET) to exhaustion performed on a cycle ergometer (Lode Excalibur Sport, Cranlea Human Performance Ltd, Birmingham, UK). Participants were asked to pedal at a consistent cadence in the range of 50–70 revolutions per minute. After a three-minute unloaded warm-up, load was progressively applied at a rate of 5–30 watts/minute based on the researcher’s estimation of their fitness level with the aim to reach maximal exertion in 8–12 min. Heart rate was recorded continuously via electrocardiogram (Vyntus, Vyaire Medical, Basingstoke, UK) and oxygen uptake by indirect calorimetry (Vmax Vyntus, Vyaire Medical, Basingstoke, UK). Every minute, RPE was reported on a 6–20 scale and blood pressure was measured using an automated sphygmomanometer. Ventilatory threshold was defined, with timepoint masked independently by two researchers, as the inflection point in the carbon dioxide production vs. oxygen uptake (V̇CO_2_/V̇O_2_) relationship and the point in which the ratio of minute ventilation to oxygen uptake (VE/V̇O_2_) systematically increased without an increase in the ratio of minute ventilation to carbon dioxide production (VE/V̇CO_2_) (Levett et al. 2018). Peak oxygen uptake (V̇O_2PEAK_) was reported as the highest 15-second average. Participants declined to complete CPET pre- and post-intervention due to knee pain when cycling (*n* = 2) and post-intervention due to claustrophobia (*n* = 1). Maximal exertion was verified by achieving age-related criteria for respiratory exchange ratio (Evardsen et al. 2014). Maximal exertion was achieved in 91% (*n* = 19/21) of pre-intervention tests and 91% (*n* = 19/21) of post-intervention tests.

#### Functional fitness

Participants were asked to complete either a 1-minute sit-to-stand test or 1-minute seated push-up test, depending on their ability to stand from a chair without using their arms. Demonstrations were provided. The sit-to-stand test was performed using a 44 cm chair and participants were instructed to stand up tall as many times as possible in one minute with their arms crossed at their chest (Bohannon and Crouch 2019). The seated push-up test is a novel functional measure to test upper limb strength without specialist equipment (Poncumhak et al. 2022, Chokphukiao et al. 2023). In our modified version, the seated push-up test was performed using a 44 cm chair with arms that extended 22 cm above the base of the chair. Participants were asked to sit in the centre of the chair with hands planted on the arms of the chair by their sides and lean their torso forward. During the test, participants were instructed to straighten their elbows to lift themselves out of the seat with minimal use of their legs as many times as possible in one minute.

### Statistical analysis

Traffic light criteria of ≤ 50%--Stop, 51–74%--Amend, and ≥ 75%--Go for adherence and retention rate were defined *a priori* by researchers and clinicians to assess whether progression to a definitive study was indicated. The Stop criterion (≤ 50%) was selected due to the binary nature of retained/dropped out and adherent/non-adherent, where if more than half of participants dropped out or were non-adherent, the likelihood of a systematic issue with the study or intervention design was deemed to preclude a definitive study. The Go criterion (≥ 75%) was selected as this cut-off has previously been used to define good adherence to prehabilitation (Alsuwaylihi et al. 2024) and allowed for expected drop-outs in the context of prehabilitation, e.g., medical issues, competing priorities. In a similar study which implemented teleprehabilitation in the NHS, adherence and retention were 72% (Wu et al. 2021). A sample size of *N* = 33–34 participants consented was estimated to provide sufficient confidence that the adherence/retention proportions we observed in this feasibility study were reflective of the total population (*α* = 0.05, ß = 0.90), minimising the risk of basing the decision to progress/not progress to a definitive study on a type I/II error (Lewis et al. 2021). We rounded the sample size up to *N* = 35. To achieve our Go criteria, minimum *n* = 27 participants were required to complete the study (retention) and ≥ 75% of ‘completers’ were required to attend 100% of exercise sessions offered (adherence). Feasibility data were reported as proportions and mean (95% confidence interval (CI)), and secondary outcomes as median (interquartile range). Data were log10 transformed to normalise data distribution prior to calculating effect sizes using the formula: *d* = ((Mean_1_–Mean_2_)÷(√((SD_1_^2^+SD_2_^2^) ÷ 2)). As physiological zeros were present within the HADS and IPAQ short-form questionnaire responses, a small constant (+ 1) was added to all raw data points for those datasets prior to log10 transformation. The magnitude of pre-intervention to post-intervention change to secondary outcomes were interpreted as *d* = 0.2 (small), *d* = 0.5 (moderate), *d* = 0.8 (large).

## Results

### Participant characteristics

**Fig. 3:**
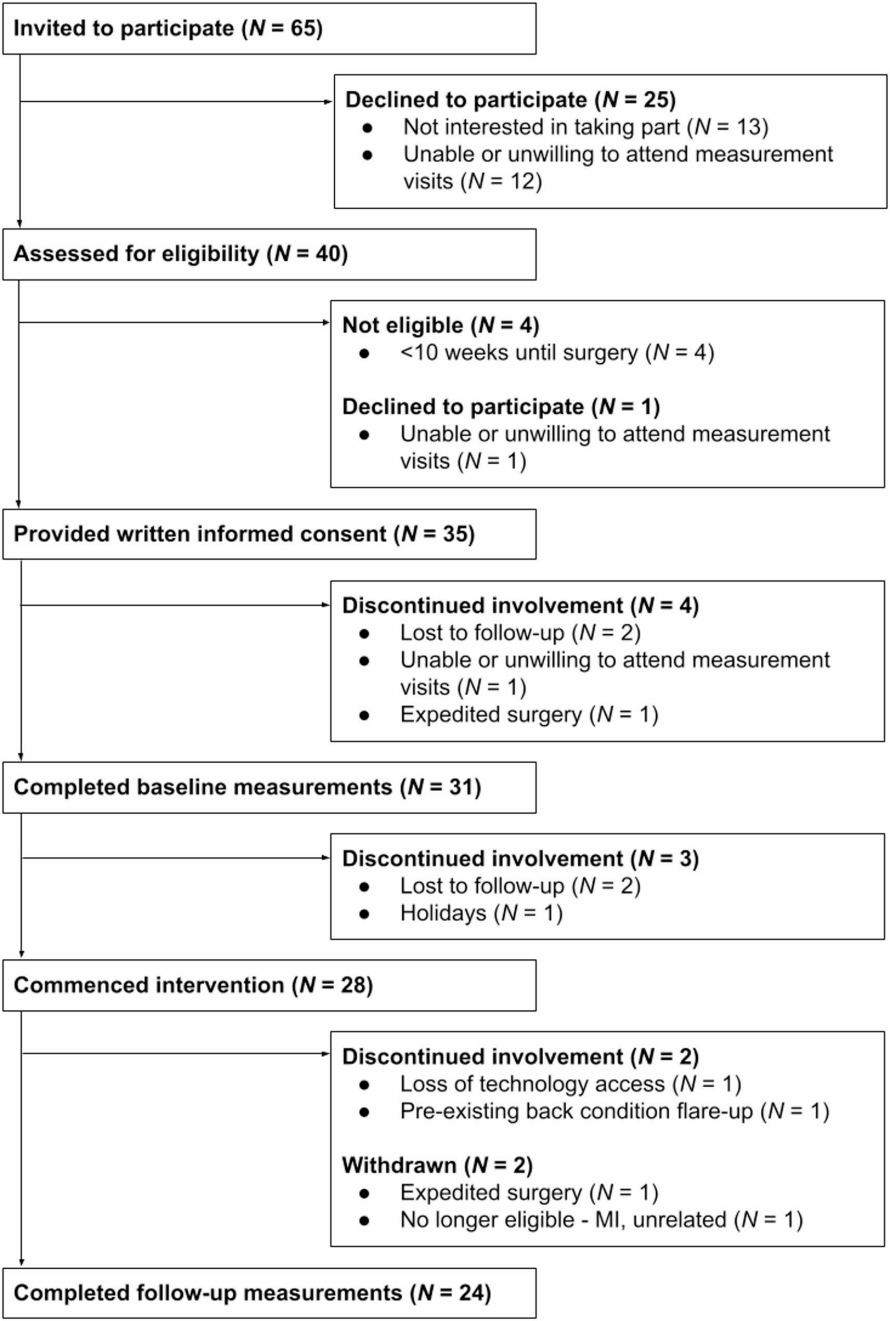
Flow diagram showing participant flow through the study

Recruitment spanned February 2024 to June 2024. Uptake rate was 65%, screen-pass rate was 90%, and overall recruitment rate was 54% (Fig. 3). Reasons for declining to participate were lack of interest (*n* = 13) and inability or unwillingness to travel to in-person measurement visits (*n* = 12) (Fig. 3). Of *N* = 35 participants that provided informed consent, participant characteristics are available for *n* = 31 participants who completed baseline measurements, and are separated into those who completed the study and those who later withdrew (Table 1). Participants who completed the study ranged in age from 53 to 86 years, and were, on average, younger than those (*n* = 7) who withdrew their participation. After baseline measurements, *n* = 3 participants withdrew before commencing the intervention, and *n* = 4 participants withdrew during the intervention (Fig. 3). Participants who dropped out during the intervention (*n* = 2) completed one and two exercise sessions, respectively. Participants who were medically-withdrawn during the intervention (*n* = 2) completed seven and 11 exercise sessions, respectively. A total of *n* = 24 participants completed follow-up measurements, representing 69% retention, falling within the ‘Amend’ range of progression criteria (51–74%). If participants who withdrew from the study due to expedited surgery were considered to have completed their involvement, the retention rate rose to 74%. The retention rate for those who commenced the intervention was 86% (Fig. 3). The *n* = 24 retained participants are discussed herein.

**Table 1:** Participant characteristics

		Participants that completed the study (n = 24)	Participants that withdrew from the study (n = 7)
Age (years)		69 (65, 75)	78 (69, 80)
Body mass index (kg/m^2^)		29.1 (23.9, 31.9)	30.2 (25.8, 31.7)
Number of medical conditions		3 (2, 4)	3 (3, 3)
Number of medications		5 (3, 9)	3 (3, 5)
Sex	Female (*n*, %)	14, 58%	2, 29%
	Male (*n*, %)	10, 42%	5, 71%
Ethnicity	White British (*n*, %)	24, 100%	6, 86%
	Mixed or multiple (*n*, %)	-	1, 14%
Surgery	Knee replacement (*n*, %)	3, 13%	4, 57%
	Hip replacement (*n*, %)	3, 13%	1, 14%
	Parathyroidectomy (*n*, %)	3, 13%	1, 14%
	Hernia repair (*n*, %)	3, 13%	-
	Thyroidectomy (*n*, %)	2, 8%	1, 14%
	Vaginal prolapse repair (*n*, %)	2, 8%	-
	Hysterectomy (*n*, %)	2, 8%	-
	Knee revision (*n*, %)	1, 4%	-
	Hip revision (*n*, %)	1, 4%	-
	Cholecystectomy (*n*, %)	1, 4%	-
	Prostatectomy (*n*, %)	1, 4%	-
	Ovarian cystectomy (*n*, %)	1, 4%	-
	Ureteral reimplantation (*n*, %)	1, 4%	-

Data are median (IQR) unless otherwise indicated. *n* = 4 participants withdrew prior to baseline measurements, so participant characteristics were not collected

### Adherence

In relation to pre-defined progression criteria for adherence, 21 of 24 participants (88%) who completed the study completed all exercise sessions, meeting our ‘Go’ criteria of ≥ 75% adherence. Participants completed 98% (95% CI 94–101) of the 18 available sessions. Participants completed three sessions every 7 days (95% CI 6–8) with 2 rest days (95% CI 2–3) between each session. Three sessions were completed within a seven-day period 76% (95% CI 65–87) of the time, and the remaining were completed in eight days (7%, 95% CI 4–10), nine days (4%, 95% CI 1–6), or ≥ 10 days (14%, 95% CI 4–23). Health reasons unrelated to the intervention (*n* = 6, summarised in ‘Safety’ below), holidays (*n* = 5), temporary technical issues (*n* = 5), bereavement (*n* = 1), and work commitments (*n* = 1) caused interruptions which extended the period within which three exercise sessions were completed beyond seven days. Total programme duration was 51 days (95% CI 44–59).

### Compliance

The mean proportion of compliant exercise sessions – performed as prescribed in terms of intensity, type, and duration of exercise – was 79% (95% CI 68–90). Exercise sessions were categorised as non-compliant due to not achieving vigorous intensity (1%, 95% CI 0–3), issues with one specific exercise (9%, 95% CI 1–17), or multiple exercises (4%, 95% CI 0–8), or not completing the full exercise duration (7%, 95% CI 0–14). Aerobic exercise was performed at RPE 8 (95% CI 7–8) and resistance exercise was performed at RPE 8 (95% CI 7–8).

### Safety

Two serious adverse events occurred during the study, which were unrelated to the intervention. One participant was admitted to hospital with diverticulitis (grade 2), and then continued to complete the intervention. One participant was successfully treated for myocardial infarction (grade 3), with onset of symptoms whilst at work, and was withdrawn from the study. Non-serious adverse events which were unrelated to the intervention were: infectious illness (*n* = 2), injury at work (*n* = 2), flare-up of fibromyalgia symptoms (*n* = 1), and diverticulitis (*n* = 1).

### In-platform fitness assessments

**Fig. 4:**
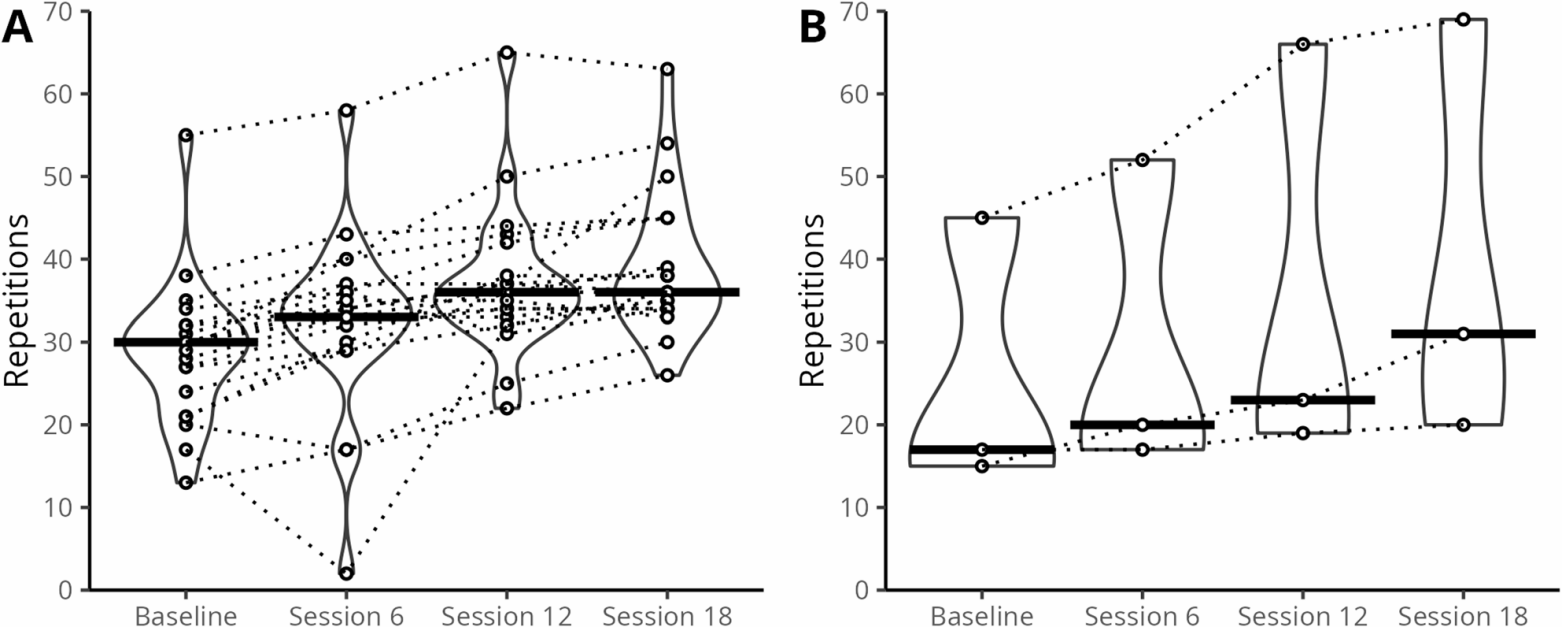
Changes to unsupervised functional fitness test scores during the intervention. Violin plot showing: **A **number of repetitions performed in an unsupervised 1-minute sit-to-stand test completed within the digital prehabilitation platform at baseline (*n* = 21), after six exercise sessions (*n* = 21), 12 exercise sessions (*n* = 20), and 18 exercise sessions (*n* = 20). **B **number of repetitions performed in an unsupervised 1-minute seated push-up test completed within the digital prehabilitation platform at baseline, after six exercise sessions, 12 exercise sessions, and 18 exercise sessions (all *n* = 3). Bold horizontal lines show the median. Open circles linked with dotted lines show individual responses

The median (IQR) unsupervised 1-minute sit-to-stand scores (*n* = 21) were 30 (24, 32) repetitions at baseline, 33 (29, 36) repetitions after six exercise sessions, 36 (33, 39) repetitions after 12 exercise sessions, and 36 (34, 45) repetitions after 18 exercise sessions (Fig. 4A). Unsupervised 1-minute seated push-up test scores (*n* = 3) were 17 (16, 31) repetitions at baseline, 20 (19, 36) repetitions after six exercise sessions, 23 (21, 45) repetitions after 12 exercise sessions, and 31 (26, 50) repetitions after 18 exercise sessions (Fig. 4B).

### Acceptability

Feedback questionnaire responses were collected from twenty participants who completed the study, and quotes were extracted. For example, one participant wrote: “*I really enjoyed the programme and found it hugely motivating. The first 2 sessions I found very easy but then it started to get harder at week 3 and then really quite challenging at week 5. I will definitely be continuing with regular exercise*,* I feel so much better in myself*,* both physically and mentally.*” Likert scale questionnaire responses and themes identified during analysis of open-ended feedback quotes are summarised in Table 2.

**Table 2:** Feedback after completing the digital prehabilitation programme

	N/A	Strongly disagree	Disagree	Unsure	Agree	Strongly agree
I would recommend the programme to a friend if they were having surgery					25%	75%
I am satisfied with the prehabilitation website				5%	30%	65%
I feel that the prehabilitation programme helped me to prepare for surgery				10%	35%	55%
The exercises in the programme felt appropriate to me				10%	55%	35%
I enjoyed doing exercises at home				10%	40%	45%
The exercise videos were easy to follow					25%	75%
I felt motivated to follow the exercise videos				5%	35%	60%
It was easy to complete the fitness tests in the programme			10%	20%	35%	35%
I felt motivated to follow the guidance in the prehabilitation eBook	20%			5%	50%	25%
The community forum was a good source of support	20%			55%	20%	5%

Themes identified from written feedback						
Enjoyable & worthwhile	Overall, participants experienced the programme as something that was very worthwhile. They considered there to be many benefits to engaging in the programme allowing them to experience further secondary benefits (e.g., reduction in pain, increase in mental health/well-being) which made the programme even more worthwhile. Generally, participants perceived the activities in the programme as enjoyable.					
Practicalchallenges to engage with the programme	Practical challenges were experienced by participants which prevented them from engaging with the programme. This included challenges such as fitting the exercises around their daily tasks/schedules, and considering how to continue the programme when going on holiday and/or not being near a technical device.					
Frustrations due to technical issues & capabilities	While the participants recognised the excellent support the team provided, they experienced technical difficulties which they mentioned were particularly frustrating. Furthermore, participants identified frustration when they were unable to use certain technical capabilities they might have expected (e.g., being able to skip the instructions of videos).					
Concerns and challenges to perform the exercises	Overall, participants identified that certain physical health problems could prevent them from engaging in the programme. (e.g., illness such as flu, injuries, feeling exhausted, other health difficulties). They also identified that they were particularly concerned about performing exercises when experiencing physical issues or injuries (although the similarity of the exercises to NHS and physiotherapeutic exercises managed to reassure most people).					
Timing of the programme	While most participants mentioned that they experienced a benefit from engaging in the programme, some participants were concerned about the benefit – particularly when the programme did not align with their surgery date. Furthermore, participants were concerned about losing out on the benefit when stopping the programme (e.g., due to still not having a date for their surgery when the programme had finished).					

Fifteen participants posted a total of 31 comments on the community forum, with 2 (1, 3) posts per participant, which were typically 2 (2, 3) sentences long. Participants posted about their progress through the programme, enjoyment of exercise sessions, perceived benefits, and challenges. For example: *“Good workout today could feel my muscles working. Just been to buy new weights I like incorporating them. Eager to push on.”* Participants also posted seeking support from the technical or prehabilitation teams, and received replies. There was no evidence of engagement between participants within the forum (i.e., replies on comments from other people taking part in the study).

### Secondary outcomes

**Fig. 5:**
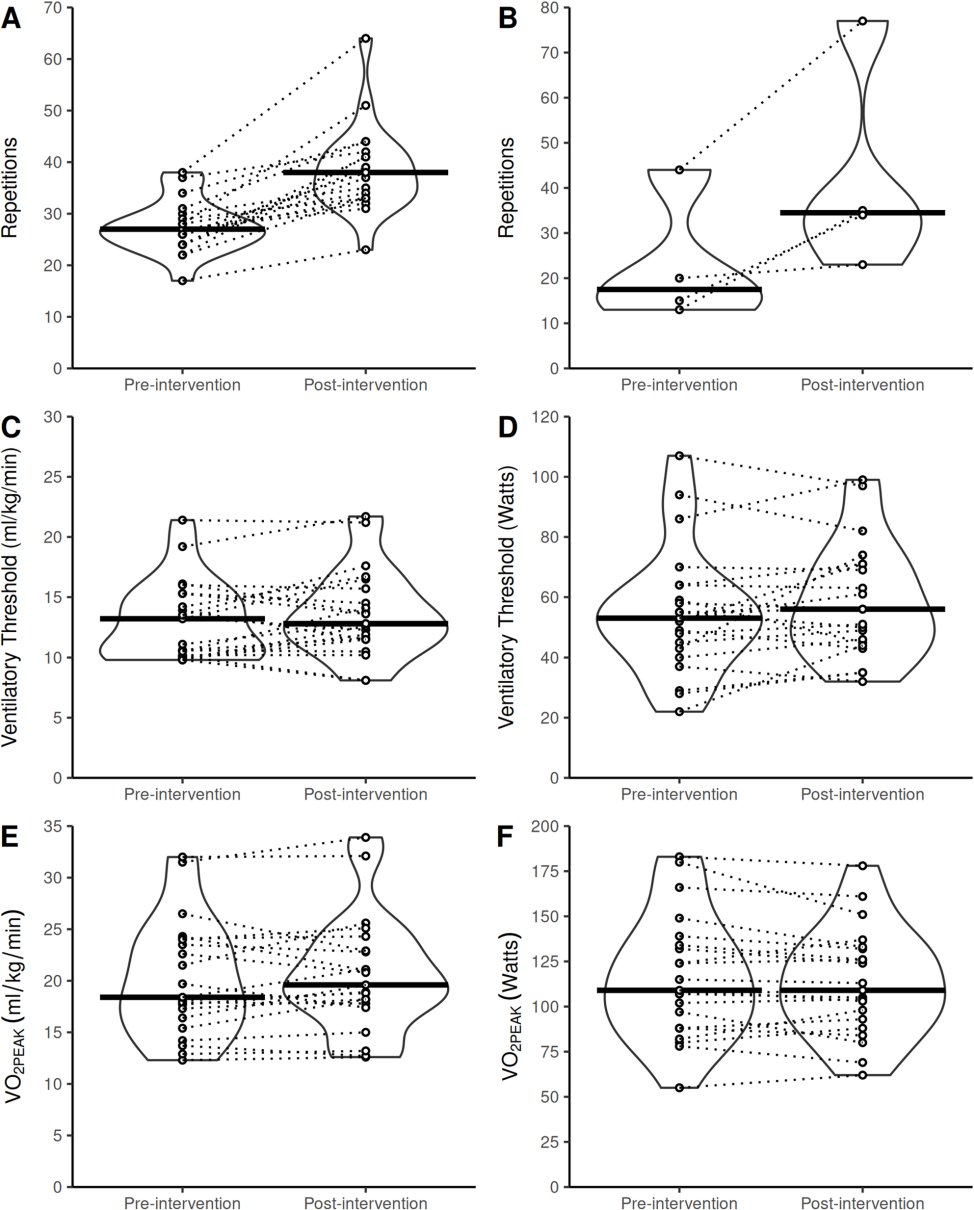
Individual changes to cardiorespiratory and functional fitness from pre- to post-intervention. Violin plots showing pre-intervention to post-intervention changes to: **A **supervised 1-minute sit-to-stand test (*n* = 19), **B **supervised 1-minute seated push-up test (*n* = 4), **C **Ventilatory threshold (ml/kg/min, *n* = 21), **D **Ventilatory threshold (watts, *n* = 21), **E **V̇O_2PEAK_ (ml/kg/min, *n* = 21), **F **V̇O_2PEAK_ (watts, *n* = 21). Bold horizontal lines show the median. Open circles linked with dotted lines show individual responses

Secondary outcomes were measured proximal to completion of prehabilitation, and always before surgery. Due to variation in the time to complete prehabilitation (see ‘Adherence’ above), the timing of the follow-up measurement visit also varied, on average occurring in Week 8 (IQR Week 7, Week 9). Pre-intervention to post-intervention changes to cardiorespiratory fitness, functional fitness, and resting blood pressure and heart rate are shown in Table 3. Functional fitness measured by 1-minute sit-to-stand test increased by 42% (20, 58) from pre- to post-intervention. Individual changes to cardiorespiratory and functional fitness are shown in Fig. 5. At baseline, ventilatory threshold was < 11 ml/kg/min for *n* = 9 participants, indicating increased risk for postoperative complications (Moran et al. 2016). After prehabilitation, *n* = 4 participants had ventilatory threshold < 11 ml/kg/min. Where the median change to oxygen uptake at ventilatory threshold was 0.0 ml/kg/min with an effect size of *d* = 0.2, examination of the mean and standard deviation revealed an increase of 0.6 ± 2.5 ml/kg/min.

**Table 3:** Change to fitness and resting observations from pre- to post-intervention

Measurement	Baseline	Follow-up	Change	Effect size
V̇O_2PEAK_ (ml/kg/min, *n* = 21)	18.4 (16.4, 24.0)	19.6 (17.8, 22.9)	0.3 (-0.7, 1.2)	0.1
V̇O_2PEAK_ (W, *n* = 21)	109 (88, 134)	109 (93, 132)	-4 (-8, 2)	0.1
Ventilatory threshold (ml/kg/min, *n* = 21)	13.2 (10.2, 15.3)	12.8 (11.5, 15.7)	0.0 (-1.0, 2.5)	0.2
Ventilatory threshold (W, *n* = 21)	53 (43, 64)	56 (45, 71)	3 (-5, 7)	0.2
1-minute sit-to-stand test (*n* = 19)	27 (24, 30)	38 (33, 42)	11 (6, 14)	1.7
1-minute seated push-up test (*n* = 4)	18 (15, 26)	35 (31, 46)	21 (15, 25)	1.2
Systolic blood pressure (mmHg)	129 (119, 144)	131 (120, 140)	1 (-10, 12)	0.0
Diastolic blood pressure (mmHg)	79 (71, 86)	81 (74, 84)	1 (-3, 5)	0.1
Resting heart rate (beats/min)	72 (65, 77)	70 (62, 75)	-2 (-7, 4)	0.0
Body mass (kg)	85.6 (64.2, 93.9)	83.3 (64.2, 92.9)	-0.4 (-1.1, 0.2)	0.0

Data are median (IQR). *n* = 24 unless otherwise indicated. ‘Change’ is the median (IQR) of individual changes. V̇O_2PEAK_ = Peak oxygen uptake. *n* = 1 participant completed a 1-minute seated push-up test at baseline and a 1-minute sit-to-stand test at follow-up, so was not included in analysis. *n* = 3 participants did not complete CPET, so were not included in V̇O_2PEAK_ and ventilatory threshold analysis

**Fig. 6:**
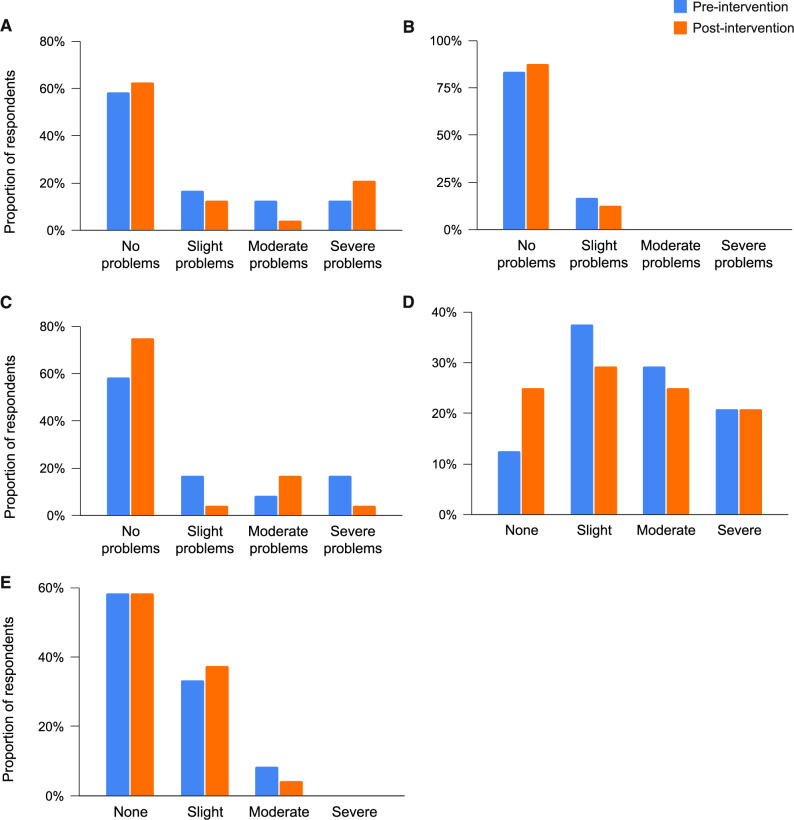
Distribution of quality of life metrics pre- and post-intervention. Proportion of respondents within each level of the EQ-5D-5L questionnaire for: **A **Mobility, **B **Self care, **C **Usual activities, **D **Pain/discomfort, and (**E**) Anxiety and depression. No participants scored ‘unable’ (**A**-**C**) or ‘extreme’ (**D**, **E**) at either measurement time-point

Pre-intervention to post-intervention changes to patient-reported outcome measures of quality of life, anxiety, depression, and typical physical activity are shown in Table 4. The distribution of EQ-5D-5L responses pre-intervention and post-intervention are shown in Fig. 6. There were increases to the proportion of participants reporting ‘no problems’ with mobility, self-care, and usual activities, and ‘none’ when asked about pain/discomfort, from pre-intervention to post-intervention (Fig. 6A-D). However, there was also an increase to the proportion of participants reporting severe mobility problems from pre-intervention to post-intervention (Fig. 6A).

**Table 4:** Change to patient-reported outcome measures from pre- to post-intervention

Measurement	Baseline	Follow-up	Change	Effect size
EQ-5D-5L VAS	70 (65, 80)	80 (70, 85)	5 (0, 15)	0.5
HADS-Anxiety	6 (5, 8)	6 (4, 7)	-1 (-2, 0)	0.2
HADS-Depression	4 (2, 5)	3 (1, 4)	-1 (-1, 0)	0.4
Sitting time (hours/day)	5.5 (4.0, 6.1)	4.0 (3.5, 5.6)	-0.5 (-1.0, 0.0)	0.3
Vigorous PA (minutes/week, *n* = 23)	0 (0, 100)	125 (30, 203)	60 (3, 135)	0.9
Moderate PA (minutes/week, *n* = 23)	0 (0, 145)	135 (0, 360)	60 (-5, 240)	0.4
Walking PA (minutes/week, *n* = 22)	255 (120, 465)	210 (98, 420)	-35 (-203, 45)	0.1
Total PA (MET-minutes/week, *n* = 20)	1241 (649, 3170)	3153 (1161, 5393)	852 (-53, 2292)	0.2

Data are median (IQR). ‘Change’ is the median (IQR) of individual changes. *n* = 24 unless otherwise indicated. HADS = Hospital Anxiety and Depression Scale; MET = Metabolic equivalent of task; PA = Physical activity; VAS = Visual analogue scale. *n* = 4 participants answered ‘not sure’ on IPAQ-short form, so did not provide data for calculation of total PA in MET-minutes/week, and vigorous or moderate or walking PA

### Carbon emissions

Twenty participants reported usually travelling to hospital by car. Participants who travelled by bus (*n* = 3) or walking (*n* = 1) were considered to have zero travel-associated carbon emissions. The median estimated travel-associated carbon emissions avoided by completing a home-based intervention compared to an equivalent hospital-based intervention was 44 (13, 63) kg CO_2_ per participant (*n* = 24), totalling an estimated 1.22 tonnes CO_2_.The median round-trip distance travelled by car per hypothetical visit to the hospital was 20 (8, 23) km and ranged from 3 to 64 km.

## Discussion

This study demonstrated the feasibility and acceptability of a novel, tailored digital exercise prehabilitation intervention (‘PreActiv’), meeting predefined criteria to support progression to a definitive trial. Overall, participants awaiting major surgery found digital prehabilitation enjoyable and worthwhile, and were consistently able to exercise at vigorous intensities without supervision and without exercise-related adverse events.

The personalisation of exercise sessions plus autonomy in scheduling may explain the excellent adherence rate, with 88% of participants fully-adherent and completion of 98% of available sessions on average in this study, which is similar (Franssen et al. 2022) or superior (Piraux et al. 2020, Singer et al. 2018) to adherence reported in other similar unsupervised digital prehabilitation studies. Adherence was also higher with this digital exercise prehabilitation programme than in previous studies of traditional home-based prehabilitation delivered via printed booklets or DVDs (Nielsen et al. 2010, Howard et al. 2019). Comparable adherence to relevant face-to-face supervised prehabilitation interventions (e.g., offering a similar exercise dose to a related patient group) (Marchand et al. 2021, McKay et al. 2012, Rooks et al. 2006) indicates that this novel digital exercise prehabilitation programme may have similar efficacy to supervised prehabilitation (Kraemer et al. 2022). A definitive study that is adequately powered to assess meaningful improvements to postoperative outcomes is required to confirm this.

High intensity interval training is a time-efficient exercise prescription which is favourable for prehabilitation due to potentially short timeframes between diagnosis and surgery (Weston et al. 2016). However, a limitation of unsupervised high intensity interval training is the observation that individuals commonly undershoot the prescribed intensity (Ekkekakis and Biddle 2023). In the present study, vigorous intensity exercise – defined as RPE ≥ 5/10 (Arney et al. 2019, Garber et al. 2011) – was performed in 99% of exercise sessions, demonstrating the utility of unsupervised high intensity interval training when delivered digitally with progressive exercises which are tailored to fitness level. The compliance to intensity herein was superior to a hospital-based, fully supervised, vigorous intensity cycling intervention, where only 30% of exercise intervals were performed at the target RPE of 5–7/10 (Tew et al. 2017). Attainment of high exercise intensities is relevant in the preoperative period as prehabilitation performed at high intensity appears to be more effective than moderate intensity at improving fitness prior to surgery (Franssen et al. 2022). Objective measurement of exercise intensity using wearable devices is warranted in a future study to better understand the importance of achieving high intensities to improve physiological outcomes related to postoperative complications, such as V̇O_2PEAK_ and ventilatory threshold.

The retention rate of 69% in the present study is comparable to other unsupervised digital prehabilitation interventions implemented within the UK NHS, where retention rates of 72% (Wu et al. 2021) and 68% (Kadiri et al. 2019) were reported. Previous supervised prehabilitation studies had similar (Rooks et al. 2006) or higher (Marchand et al. 2021, McKay et al. 2012, Barakat et al. 2016) retention rates than the present study, which may be explained by increased accountability with person-person interactions, rather than interactions with a digital platform. Indeed, loss to follow-up was the most common reason for drop-out in the present study (11%), which may be due to lower accountability with fewer personal interactions. Other reasons for withdrawal included: expedited surgery (6%), health reasons (6%), holidays (3%), loss of technology access (3%), and in-person measurement visits (3%). Tailoring the prehabilitation programme to anticipated surgery date would address the issue of defining retention in the case of expedited surgery, and also improve satisfaction with the programme, as the asynchronous timing of the prehabilitation programme in relation to surgery was identified as a concern in participant feedback. Recent technical developments to ‘PreActiv’ allow programme length to be dynamically adjusted based on predicted surgery date entered by patients or autopopulated via integration with other digital healthcare systems. Leveraging this progress in future studies may improve patient satisfaction and benefit.

Recruitment rate in the present study was impeded by the in-person research measurement visit, which reflects the challenges posed by travelling to appointments in this group, and further rationalises a digital, home-based prehabilitation programme. Substituting the in-person measurement visit with remote measurements in a future study may improve recruitment and retention, whilst also reducing carbon emissions, and providing a sustainable method – with lower financial and time cost – for ongoing evaluation of the platform outside of the context of a clinical trial. Other participants who declined participation were uninterested in the study, but did not provide a more specific reason. Uncertainty about engaging in exercise training with preexisting medical conditions was raised as a concern in feedback from participants who completed the study, and may also have been a barrier to participation for some patients. As such, the suitability of exercises with varying physical health conditions should be explained in more detail in the participant information sheet to improve recruitment rate in a future study. We did not provide technology to facilitate participation in this study, which may introduce bias via digital exclusion. Ongoing engagement with groups at risk of digital exclusion will continue to improve the accessibility of the novel prehabilitation platform. Future studies should recruit within equity deserving groups, and support their engagement via provision of devices and support with digital skills, to evaluate whether the digital prehabilitation programme is effective for a diverse sample.

An important goal of prehabilitation is to enhance fitness, due to associations between fitness level and risk of postoperative complications. Indeed, oxygen uptake at ventilatory threshold < 9–11 mL/kg/min and peak effort < 15 mL/kg/min are associated with increased risk of morbidity and mortality after major surgery (Moran et al. 2016). While the present study was not powered to assess changes to cardiorespiratory fitness, it is notable that ~ 40% of participants had ventilatory threshold < 11 ml/kg/min at baseline, reducing to 20% of participants at follow-up, indicating potential for clinically-significant improvement. Extending the prehabilitation programme length by tailoring to surgery date may provide more opportunity to improve V̇O_2PEAK_ and ventilatory threshold. Large improvements to functional fitness were apparent after the prehabilitation programme, which are likely attributable to the high levels of adherence and compliance to high intensity interval training. There were also large increases to self-reported physical activity, however these findings should be interpreted with caution, as IPAQ short-form has been known to overestimate physical activity level (Lee et al. 2011) and there was missing data for IPAQ short-form in our dataset. There were indications for improved quality of life, anxiety, and depression from pre-intervention to post-intervention, evident via small to moderate effect sizes. The digital intervention included generic written advice on psychological support strategies within an eBook, but it is notable that many participants did not access this information. As such, the benefits are likely attributable to exercise training, in line with prior evidence that exercise is an effective treatment for anxiety and depression (Rebar et al. 2015). Although, it should be acknowledged that involvement in a study of any kind may improve mental health due to more frequent healthcare interactions. Randomised controlled trial designs will be required to evaluate whether participation in digital prehabilitation improves mental preparedness for surgery.

This study has numerous strengths. The exercise prescription was developed considering best practice guidelines for prehabilitation (Tew et al. 2018). The use of in-platform fitness tests after completing six, 12, and 18 sessions (equivalent to testing every two weeks for participants who completed three sessions per week) to guide progression of the exercise prescription is a prominent strength of this programme, with lack of objectively defined progression identified as a downfall of existing supervised and unsupervised prehabilitation programmes (Thomas et al. 2019). Furthermore, feasibility and acceptability of the intervention was evaluated using both quantitative and qualitative data, providing a rich picture of engagement, fidelity, and acceptability amongst participants. In particular, a meaningful assessment of adherence was possible due to requirements to watch videos to completion and submit feedback before a session was considered complete. The present study also has design limitations. Completion of the prehabilitation programme did not always coincide with surgery date, causing some dissatisfaction for participants relating to the risk of reversibility in fitness adaptations. The feasibility of randomisation was not evaluated in this study, meaning recruitment and retention rates in a definitive randomised-controlled trial may differ from those observed here. The lack of control group and small sample size precludes our ability to definitively attribute any effects on fitness, health, and wellbeing to the intervention. Additionally, postoperative outcomes were not assessed to give an indication of the longer term impact of the intervention, but would be a valuable outcome in any ensuing study.

## Conclusions

This single-armed study demonstrated the feasibility and acceptability of a novel, tailored, and progressive digital exercise prehabilitation intervention for patients awaiting major elective surgery. Progression criteria for retention and adherence were achieved, prehabilitation was performed with high compliance, and there were no related serious adverse events. Accordingly, future collaborative randomised-controlled trials – involving multiple sites and diverse samples – exploring the effects of the ‘PreActiv’ digital prehabilitation on postoperative outcomes are both promising and justifiable.

## Data Availability

The datasets used and/or analysed during the current study are available from the corresponding author on reasonable request.

## Abbreviations

BMI: Body mass index
CI: Confidence interval
CPET: Cardiopulmonary exercise test
HADS: Hospital anxiety and depression scale
IPAQ: International physical activity questionnaire
IQR: Interquartile range
MET: Metabolic equivalent of task
NHS: National Health Service
PA: Physical activity
RPE: Rating of perceived exertion
VAS: Visual analogue scale
V̇O_2PEAK_: Peak oxygen uptake
